# Highly efficient and safe genome editing by CRISPR-Cas12a using CRISPR RNA with a ribosyl-2′-O-methylated uridinylate-rich 3′-overhang in mouse zygotes

**DOI:** 10.1038/s12276-020-00521-7

**Published:** 2020-11-09

**Authors:** Dae-In Ha, Jeong Mi Lee, Nan-Ee Lee, Daesik Kim, Jeong-Heon Ko, Yong-Sam Kim

**Affiliations:** 1 Genome Editing Research Center, KRIBB, Daejeon, 34141 Republic of Korea; 2 KRIBB School of Bioscience, Korea University of Science and Technology (UST), 34141 Daejeon, Republic of Korea; 3 GenKOre, Daejeon, 34141 Republic of Korea

**Keywords:** Gene targeting, Genetic engineering

## Abstract

The CRISPR-Cas12a system has been developed to harness highly specific genome editing in eukaryotic cells. Given the relatively small sizes of Cas12a genes, the system has been suggested to be most applicable to gene therapy using AAV vector delivery. Previously, we reported that a U-rich crRNA enabled highly efficient genome editing by the CRISPR-Cas12a system in eukaryotic cells. In this study, we introduced methoxyl modifications at C2 in riboses in the U-rich 3′-overhang of crRNA. When mixed with Cas12a effector proteins, the ribosyl-2′-O-methylated (2-OM) U-rich crRNA enabled improvement of dsDNA digestibility. Moreover, the chemically modified U-rich crRNA achieved very safe and highly specific genome editing in murine zygotes. The engineered CRISPR-Cas12a system is expected to facilitate the generation of various animal models. Moreover, the engineered crRNA was evaluated to further improve a CRISPR genome editing toolset.

## Genome editing: chemical change improves CRISPR tool

A chemical modification to the CRISPR-Cas12a gene-editing system could allow for safer and more efficient genome editing. Yong-Sam Kim and colleagues from the Korea Research Institute of Bioscience & Biotechnology (KRIBB) in Daejeon, South Korea, chemically altered the RNA molecule that helps guide Cas12a enzymes to their target genome sequences, where the CRISPR machinery initiates DNA cutting. Building on a previous modification, in which Kim’s team at the KRIBB Genome Editing Research Center added poly-uridinylates to the RNA, the researchers further modified the RNA with methyl tags. This increased the DNA-cutting activity of Cas12a, both in test tube experiments and in mouse embryos, resulting in safe and specific genome editing. The modifications could lead to more precise genetic models of human diseases or be incorporated into future gene therapies.

## Introduction

The clustered regulatory interspaced palindromic repeat (CRISPR) system has “democratized” genome editing technology because of its easy construction, high performance, and versatile applications in eukaryotic systems^1–4^. Since the development of the CRISPR-Cas9 system, various CRISPR systems have been identified in bacteria and archaea, including CRISPR from *Prevotella* and *Francisella* 1 (Cpf1, also known as Cas12a), Cas13, and Cas14 (recently classified as Cas12f); these systems constitute a diverse genome editing toolbox in which each tool has unique utility^5–7^. The CRISPR genome-editing tool consists of a gene-targeting guide RNA and a Cas endonuclease^8^. These two components form a ribonucleoprotein (RNP) complex that recognizes target sequences accompanying a protospacer-adjacent motif (PAM), subsequently inducing a double-stranded break (DSB) either inside or outside the protospacer region^8^.

Although the CRISPR system per se has shown surprisingly high genome editing performance, efforts to further improve the CRISPR system have been made by engineering both Cas nucleases and guide RNAs. Catalytically modified Cas9^7^ and Cas12a^8^ have been determined to have expanded utility for base editing^9–12^, epigenetic regulation^13,14^, transcriptional inhibition (CRISPRi)/activation (CRISPRa)^15,16^, library screening^17^, and, recently, prime editing systems^9^. In addition, sophisticated engineering of Cas variants has enabled high-fidelity^18–21^ and unrestricted gene targeting^10,11^. In addition, efforts to engineer guide RNAs have been rigorously undertaken to ameliorate technical and clinical limitations in research and gene therapy.

Plasmid DNA is the preferred material for genome editing in cell lines due to its high stability and transfection efficiency and to the ease with which its production can be scaled. However, plasmid DNA delivery is undesirable for engineering the genomes of plants and zygotes; rather, RNP is the preferred form of genome editor because of the lack of concern regarding chromosomal integration, its controlled expression and the possibility of specific gene editing^12^. Moreover, RNP delivery has an additional advantage because chemically modified guide RNAs can be used for efficient genome editing with improved efficiency and target specificity; in addition, regulation of biological toxicity, sensitive and specific molecular imaging, multiplexing, and genome editing flexibility is possible with modified guide RNAs^13^. Because guide RNAs are amenable to a variety of chemical modifications and mass production, chemically modified guide RNAs can be simply mixed with Cas proteins at determined stoichiometries prior to injection into zygotes^14–16^ or protoplasts^17,18^.

Previously, we reported that a uridinylate-rich 3′-overhang in crRNA significantly improved the genome editing efficiency of CRISPR-Cas12a in eukaryotic cells without compromising off-target activity^19^. The engineered U-rich Cas12a system was explored exclusively in eukaryotic cell lines, and crRNA was transcriptionally produced from DNA templates, indicating that a chemically unmodified, natural form of crRNA was used for all experiments. In this study, we tested several types of chemical modifications in the polyuridinylated 3′-overhang of crRNA and found that 2′-O-methylation (2-OM) in the ribose ring enabled genome editing of mouse zygotes with further improved genome editing efficiency and specificity. Furthermore, the 2-OM U-rich crRNA showed significantly reduced cellular toxicity when injected into zygotes with a microinjector. The optimized form of crRNA is expected to facilitate manipulation of zygotes and, subsequently, generation of animal models in a highly safe and efficient fashion.

## Materials and methods

### Preparation of vector constructs for in vitro cleavage assay

The human DNMT1 gene was amplified through polymerase chain reaction (PCR) using Pfu polymerase (Biofact, PD302-50h). Primers were screened with Primer3web version 4.1.0 and synthesized by Bioneer Corporation (Korea). The amplicon and primer sequences for DNMT1 are noted in Fig. S1. The PCR products were purified with a HiGene Gel & PCR Purification system (Biofact, GP104-100), and cloned into an All in One PCR Cloning vector (Biofact, VT201-020). The cloned vector constructs were extracted using an NucleoBond Xtra Midi kit (Machery-Nagel, 740410.50).

### Preparation of gRNA and Cpf1 protein

Each gRNA was designed to target the DNMT1 or Trp 53 gene with a TTTV PAM (Fig. S1). Each gRNA was synthesized by Integrated DNA Technologies, Inc. For an in vitro cleavage assay, recombinant *Acidaminococcus sp*. Cpf1 (AsCpf1) was prepared. Codon-optimized Cpf1 CDS from *Acidaminococcus sp*. was ligated into a pET-28a(+) plasmid vector (Addgene, 69864-3), and the vector was cloned to transform BL21(DE3) *E.coli* cells (Thermo Fisher, EC0114). The transformed colony was cultured at 37 °C in LB broth in the presence of 50 µg/ml kanamycin following preculture for 4 h in the same medium. When the optical density reached ca. 0.6., isopropyl β-D-1-thiogalactopyranoside (IPTG) was added at 30 mM, and culture proceeded at 30 °C overnight. After induction, *E.coli* cells were harvested by centrifugation at 3000 rpm for 30 min. The pellets were suspended in phosphate-buffered saline (PBS) with an EDTA-free protease inhibitor cocktail and lysed in a sonicator for 30 min at 30% pulse power with 2-s intervals (Sonics Materials, VCX-500). The lysates were cleared by centrifugation at 13,000 rpm for 30 min. After being filtered through a 0.22 µm filter unit, the supernatant was loaded onto a Ni^2+^-affinity column using an FPLC Purification System (ÄKTA Purifier, GE Healthcare). The concentrations of the purified proteins were determined from Coomassie blue staining of the bands on SDS-PAGE gels using bovine serum albumin (BSA) as a calibrator.

### **In vitro** digestion assay

Target-carrying plasmid vectors were digested in the presence of AsCpf1 protein (2.5–5 ng/µl) and 10 pM gRNA in 1X NEB 3.1 buffer. After digestion at 37 °C for 1 h, each sample was loaded onto a 1% agarose gel containing 5% ethidium bromide (EtBr). The band intensities were measured by illumination with ultraviolet (UV) light to calculate the levels of DNA cleavage.

### **In vivo** experiment

C57BL/6J mice were cared for and used for preparation of fertilized eggs. The procedures for the use and care of mice were reviewed and approved by the Institutional Animal Care and Use Committee (IACUC), KRIBB. All mice were bred in an isolated facility that was specific pathogen-free with a constant temperature of 24 °C, a humidity of 40%, and light cycles of 12 h. Forty-eight hours before human chronic gonadotropin (hCG) (Sigma-Aldrich, CG100) injection, 5 IU of pregnant mare serum gonadotropin (PMSG) (Daesung Microbiological Labs, A7101) was intraperitoneally injected into 5-week-old C57BL/6J female mice. Two days after mating, zygotes were collected from the ampullae of the oviducts, and the cumulus cells surrounding the zygotes were degraded by incubation in M2 medium (Sigma-Aldrich, M7167) containing 3 mg/ml hyaluronidase (Sigma-Aldrich, H3506) at room temperature for 5 min. After several washes in M2 and KSOM media (Merck, MR-121-D), the zygotes were cultured in KSOM medium for 5–6 h to select fertilized eggs. Fertilized eggs that formed polar bodies were used for RNP delivery.

### RNP delivery via electroporation

Fertilized eggs were suspended in Opti-MEM (Gibco, 31985-070) in the presence of RNP and placed on microscope slides with platinum-plated electrodes (NEPA GENE, CUY505P5). RNP was delivered into zygotes using a NEPA 21 Super Electroporator (NEPA GENE) according to the recommended protocol. Four days after incubation of the eggs in KSOM medium at 37 °C, cells at the morula stage were harvested for preparation of genomic DNA.

### Deep sequencing analysis

Morula cells were suspended in PCR tubes with blastocyst lysis buffer (BLB) containing 60 µg/ml yeast tRNA (Invitrogen, AM7119) and 125 µg/ml proteinase K (Qiagen, 1114886). To prepare crude genomic DNA, the PCR tubes were placed in a thermal cycler, incubated at 95 °C for 10 min and 56 °C for 20 min and then stored at 4 °C. Using a REPLI-g Mini Kit (Qiagen, 150025), the crude genomic DNA was PCR-amplified using KAPA HiFi HotStart DNA Polymerase (Roche) according to the manufacturer’s instructions. The resulting PCR amplicons carrying Illumina TruSeq HT dual indexes were subjected to 150-bp paired-end sequencing using an Illumina iSeq 100. The indel frequencies were calculated by MAUND, which is available at https://github.com/ibs-cge/maund.

### Selection of candidate off-target sites

To compare the specificity of each gRNA, potential off-target sites associated with a Trp53 on-target sequence were screened with the Cas-OFFinder program (http://www.rgenome.net/cas-offinder) with criteria of fewer than two bulges and three mismatches.

### Statistical analysis

Statistical tests of indel efficiency were performed in SigmaPlot software using a two-tailed Student’s *t* test. *P*-values < 0.05 were considered to indicate significance and are presented in the Legends section.

## Results

### Improvement of DNA cleavage activity of Cas12a via U-rich CRISPR RNA with 2′-O-methyl ribose

**Fig. 1:**
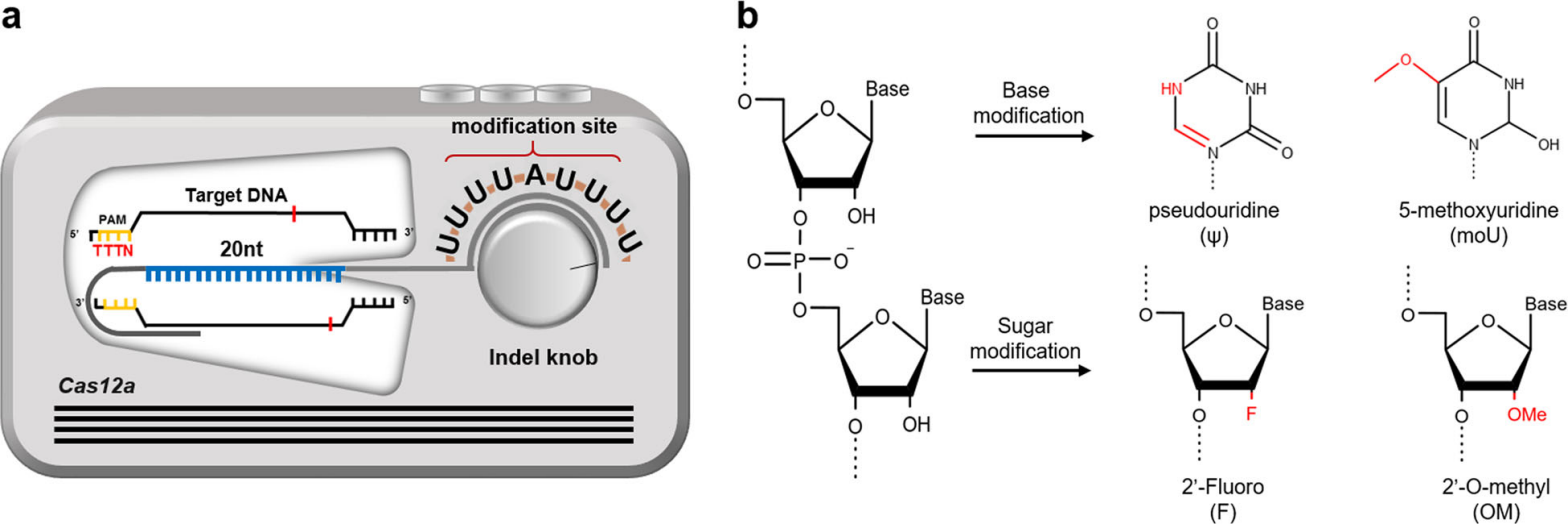
Scheme for the controllable CRISPR-Cas12a system with a 3′-polyuridinylated (U) crRNA and chemical modifications at the U-rich 3′-overhang. **a** The U-rich crRNA Cas12a system was compared to a tuner system in which the polyuridinylated 3′-overhang acts as an indel knob. By modulating the number of Us, we can regulate the indel efficiency of the CRISPR-Cas12a system in eukaryotic cells. We aim to further enhance the Cas12a system by introducing chemical modifications in the U-rich 3′-overhang. **b** Chemical modifications in crRNA were implemented in both bases and ribose sugars. The uracil base was substituted with pseudouridine (ψ) or 5′-methoxyuridine (moU). Ribose was chemically modified at C2 with a fluoro or a methyl group. The modified atom is noted in red letters.

A variety of chemical modifications have been extensively explored for Cas9 and Cas12a in the directed repeat and spacer regions of the guide RNAs. However, no experimental efforts have been made to investigate the effects of chemical modifications in the U-rich 3′-overhang region in crRNA on the functionality of the Cas12a system. Based on previous reports that modifications in bases or ribose sugars of guide RNAs improve stability and editing efficiency^20–33^, we focused on chemical modifications of bases and ribose sugars in the U-rich regions (Fig. 1a). The base modifications into nitrogen (N_5_) were made at C_5_ in the pyrimidine ring to render a pseudouridine base (ψ). The hydrogen atom attached to C_5_ was also replaced with a methoxyl group, thereby producing 5′-methoxyuridine (moU). Alternatively, the hydroxyl group at C2 in ribose was modified with a fluorine or methyl group, hereafter designated F or OM, respectively (Fig. 1b).

**Fig. 2:**
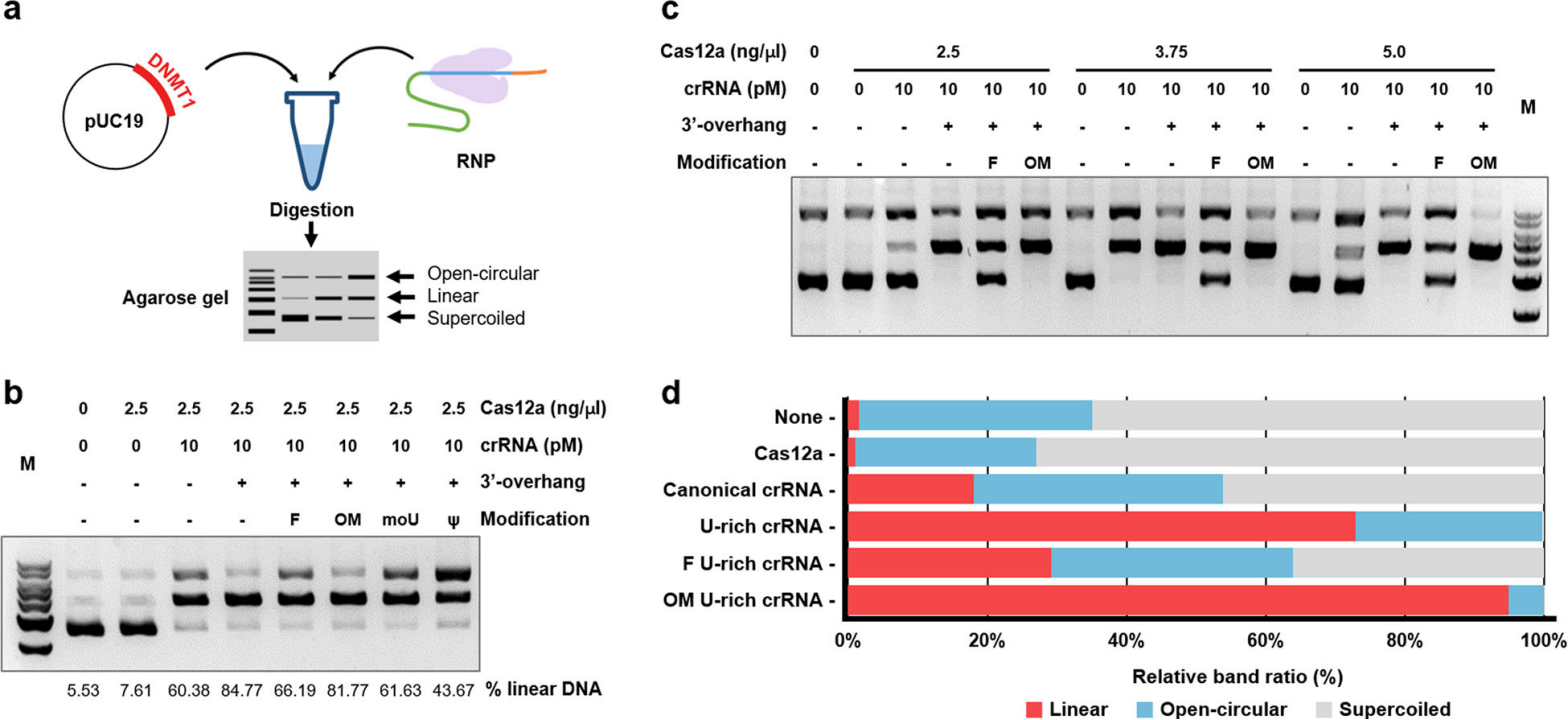
Determination of the highly efficient form of a chemical modification in guide RNA through an in vitro digestion assay. **a** Scheme for an experimental procedure to assess the efficiency of various guide RNAs. A pUC19 vector harboring the DNMT1 target sequence was digested with RNP. The crRNA was varied with sequence and/or various chemical modifications. Digested samples were run on agarose gels, on which supercoiled, linear, and open-circular forms of digested vectors were separated with different mobilities. The efficiency of the crRNAs was assessed by comparing the band intensities. **b** crRNAs with different chemical modifications (F, OM, omU, and ψ) were compared with respect to dsDNA digestibility. The guide RNA efficiency can be calculated from the band intensity ratio of an open-circular over a linear form. M refers to a 1-kb molecular ladder (lane 1). **c**–**d** The increase in Cas12a concentration caused by OM U-rich crRNA improved DNA digestibility. Increased concentrations of Cas12a promoted the digestion rate of supercoiled plasmid vectors through open-circular to linear forms. **d** In the presence of Cas12a (5.0 ng/µl) and 10 pM crRNA, compared to canonical and unmodified U-rich crRNA, OM U-rich crRNA mediated significantly better digestion efficiency.

We employed an in vitro digestion assay to assess guide RNA efficiency, in which plasmid DNA vectors harboring a 20-nt DNMT1 protospacer sequence were used as digestion substrates for the Cas12a-crRNA RNP complex (Fig. 2a). Under limited-digestion conditions, a subset of supercoiled plasmid vectors was transformed through an open circular state into linearized DNA. The ratio of linear to supercoiled forms and/or linear to open circular forms was regarded as a measure of crRNA efficiency. The incubation time and concentrations of RNP and substrate plasmid DNA were predetermined in this experiment. In line with the findings of the previous study, the U-rich crRNA significantly increased the DNA digestion activity in vitro (lane 4 vs lane 5). However, base modification of the crRNAs (ψ and moU) did not yield any increased cleavage activity (Fig. 2b). Rather, it compromised the modulatory effects of the U-rich 3′-overhangs. The F U-rich crRNA was also ineffective in modulating crRNA efficiency. In contrast, the OM U-rich crRNA and the unmodified U-rich crRNA showed comparable levels of digestion efficiency. Thus, we decided to test OM-crRNA under altered conditions. We increased the input of AsCas12a in the presence of equal amounts of crRNA and plasmid DNA but used a shortened incubation time (Fig. 2c). As the input of AsCas12a increased, a difference in cleavage activity became evident between unmodified and OM U-rich crRNA (*p* < 0.05). Notably, OM U-rich crRNA showed markedly higher efficiency than canonical crRNA (Fig. 2d). Taken together, these findings indicate that ribosyl-2′-O-methylation in the 3′-overhang of crRNA further potentiated the efficiency of Cas12a in vitro.

### Low toxicity and improved efficiency of OM U-rich crRNA for genome editing in zygotes

**Fig. 3:**
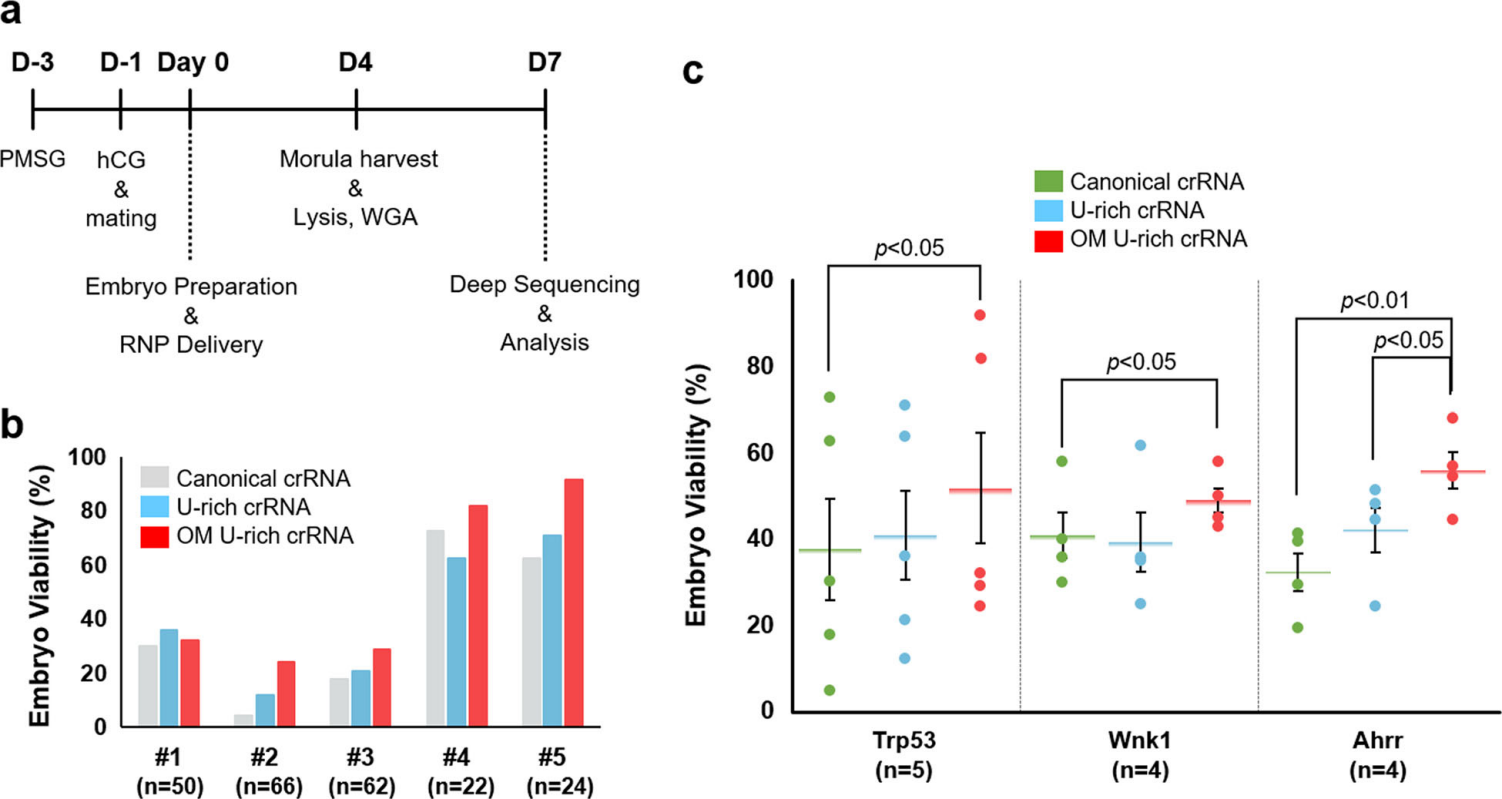
Improvement of viability and indel efficiency in embryos by OM U-rich crRNA. **a** Procedure for assessing the cellular toxicity of different types of crRNAs. Female C57BL/6J mice were injected with the hormones pregnant mare serum gonadotropin (PMSG) and human chronic gonadotropin (hCG) before mating. Zygotes were collected from the ampullae of the oviducts and treated with RNP. Forty-eight hours post-injection, morula-stage cells were counted, and genomic DNA was prepared from the viable cells. On-target and off-target loci were amplified for deep sequencing analysis. **b** Comparison of the survival rates of embryos post RNP transfection. Viability was calculated by the ratio of the number of viable embryos to the total number of transfected zygotes. The total number of zygotes for each experimental batch is presented as “n”. **c** Viability tests for multiple target genes. OM U-rich crRNA yielded more viable cells when injected into zygotes than canonical crRNA.

In addition to efficiency, toxicity is a critical issue in genome editing in zygotes because the number of zygotes available is limited. Although modification of the 5′-terminal triphosphates of guide RNAs into hydroxyl groups has been shown to improve cellular viability^26^, less is known about the effects of OM modifications, particularly in the U-rich 3′-overhang, on the viability of zygotes. To investigate the cellular toxicity of OM U-rich crRNA, we injected AsCas12a with either canonical or modified crRNAs into zygotes and monitored viability at the morula stage 4 days after injection (Fig. 3a). For this, we tested the toxicity of RNP preparations in zygotes in five independent experiments. The first three experiments (experiment 1 to experiment 3) yielded, overall, low survival rates for all the types of crRNAs. The overall low survival rates likely stemmed from the long durations of zygote manipulation due to the large number of zygotes tested (*n* = 50, 66, 62 for each type crRNA). Nonetheless, OM crRNA enabled high-viability genome editing in zygotes (Fig. 3b). To shorten the duration of zygote manipulation, we decreased the number of zygotes to 22 and 24 for experiments 4 and 5, respectively. As a result, the overall survival rate increased significantly to more than 60%. In these two experimental batches, OM U-rich crRNA was responsible for the improved viability of zygotes (Fig. 3b, Table S1). To investigate whether OM U-rich crRNA consistently shows reduced toxicity for multiple targets, we added two additional murine genes, Wnk1 and Ahrr, and investigated the cellular toxicity in murine zygotes. The results indicated that OM U-rich crRNA versus canonical crRNA was responsible for the higher viability of murine zygote cells (Fig. 3c, Table S1, S2, and S3). Thus, we concluded that U-rich crRNA with ribosyl-2′-O-methylation lowered cellular toxicity when injected into zygotes as an RNP complex. Currently, it is unclear why ribosyl modifications lower cellular toxicity, but it is tempting to speculate that the 2′-hydroxyl group in RNA is chemically reactive and that methoxyl modification might prevent unwanted intracellular molecular reactions that may lead to cellular toxicity.

Then, we investigated whether OM U-rich crRNA shows improved genome editing efficiency in zygotes as measured by a DNA cleavage assay in vitro. Because experiments 4 and 5 showed convincingly high cell viability, we investigated indel efficiency in morula cells of the two experiments. The viable morula-stage cells were pooled, and genomic DNA was prepared. Deep sequencing analysis was performed with PCR-amplified DNA products, with Trp53 as the target. As compiled in Table 1, the U-rich crRNA significantly improved indel efficiency (0.296 to 0.783 and 0.173 to 0.979 for experiments 4 and 5, respectively). OM crRNA either retained or further improved the indel efficiency of the U-rich crRNA-AsCas12a RNP complex. Notably, in experiment 4, all 18 surviving morula cells showed biallelic indel mutations (Table 2, Fig. S2). In experiment 5, OM U-rich crRNA also showed a better indel efficiency than a canonical crRNA. Although the OM crRNA was slightly less efficient than an unmodified U-rich crRNA, the overall yield of mutations was higher than that of the unmodified U-rich crRNA considering the higher viability. Because the increased indel efficiency of OM U-rich crRNA was similarly observed for the Wnk1 and Ahrr genes, we conclude that OM U-rich crRNA facilitates the generation of genome-engineered mouse models with increased safety and efficiency. Further study is needed to determine whether the improved functionality of the engineered crRNA is applicable to zygotes of animals other than mice.

**Table 1:** Increased indel efficiency of AsCas12a mediated by U-rich crRNA with OM modification in zygotes.

Experiment	Trp53 (Experiment #4)			Trp53 (Experiment #5)			Wnk1			Ahrr		
	*N* = 22			*N* = 24			*N* = 20			*N* = 30		
	Viability		Indel (%)	Viability		Indel (%)	Viability		Indel (%)	Viability		Indel (%)
	# of viable cells	%		# of viable cells	%		# of viable cells	%		# of viable cells	%	
Canonical crRNA	16	72.73	29.24	15	62.50	17.26	6	30.00	77.15	11	36.67	13.59
U-rich crRNA	14	63.64	77.96	17	70.83	97.88	5	25.00	79.67	14	46.67	27.25
OM U-rich crRNA	18	81.82	99.62	22	91.67	81.64	10	50.00	84.38	19	63.33	36.50

**Table 2:** Preserved high specificity of AsCas12a with OM U-rich crRNA.

Site	crRNA Type	#4				#5			
		Read Counts			Indel Frequency (%)	Read Counts			Indel Frequency (%)
		WT	Indel	Total		WT	Indel	Total	
**On Target**	none	9522	24	9546	0.25	9440	30	9470	0.32
	canonical	6516	2692	9208	29.24	7852	1638	9490	17.26
	U-rich	2073	7334	9407	77.96	180	8303	8483	97.88
	OM U-rich	37	9772	9809	99.62	1493	6640	8133	81.64
**OF1**^**a**^	none	541	276	817	33.78	256	77	333	23.12
	canonical	555	270	825	32.73	593	299	892	33.52
	U-rich	657	266	923	28.82	421	168	589	28.52
	OM U-rich	344	186	530	35.09	146	55	201	27.36
**OF2**	none	10503	0	10503	0	9337	0	9337	0
	canonical	10562	0	10562	0	10433	2	10435	0.02
	U-rich	11181	0	11181	0	9467	3	9470	0.03
	OM U-rich	9104	3	9107	0.03	8945	0	8945	0
**OF3**	none	11137	7	11144	0.06	9224	0	9224	0
	canonical	11173	0	11173	0	10844	0	10844	0
	U-rich	11337	14	11351	0.12	10818	0	10818	0
	OM U-rich	11041	0	11041	0	8487	0	8487	0
**OF4**	none	9676	0	9676	0	10360	6	10366	0.06
	canonical	5148	5456	10604	51.45	6595	3482	10077	34.55
	U-rich	5304	5517	10821	50.98	6022	3571	9593	37.23
	OM U-rich	5758	3748	9506	39.43	3070	5695	8765	64.97
**OF5**	none	11740	0	11740	0	10100	8	10108	0.08
	canonical	7659	0	7659	0	11346	0	11346	0
	U-rich	11762	0	11762	0	10793	0	10793	0
	OM U-rich	10577	0	10577	0	8702	0	8702	0

^a^OF1 carries a polyguanidylated sequence, and most reads had sequencing errors in the poly-G region. Manual reading confirmed the absence of reads with indel mutations outside the polyguanidylated region. The OF1 sequence is “TTTCCTTCCATGCAGATAAGATGGGGGGGGGGGC”.

### Preserved high specificity of Cas12a by OM U-rich crRNA

Despite the improved indel efficiency and mitigated cellular toxicity of OM U-rich crRNA, there remained a concern that the engineered guide RNA might impair the innately high specificity of Cas12a, thereby compromising the utility of the engineered CRISPR–Cas12a system. To investigate this possibility, we measured off-target effects in a targeted manner. We searched for potential off-target sites for Trp53 using the Cas-OFFinder program with criteria of fewer than three mismatches and two bulges. We screened five potential sites (Table S4) and performed deep sequencing analysis using genomic DNA prepared from the morula-stage cells of the previous experiments 4 and 5.

As shown in Table 2, OM U-rich crRNA did not significantly affect the off-target activity for four (OF1, OF2, OF3, and OF5) of the five potential off-target sites (Table 2). It appeared that OF1 showed a high indel frequency independent of the type of crRNA. However, we found that it originated from sequencing errors because OF1 has a polyguanylated sequence immediately downstream of the protospacer sequence. We manually investigated the sequencing data and found that, apart from the poly-G motif, few indel mutations were found for the three crRNAs tested. One intriguing result was observed for OF4, which carried a sequence almost identical to that of the target site with a single nucleotide mismatch. Moreover, the mismatch resided in an area with a high mismatch tolerance in the CRISPR-Cas12a system^34,35^. For OF4, compared to canonical and unmodified U-rich crRNA, OM crRNA increased the level of indel mutations in one experimental batch (experiment 5). We hypothesize that the increased indel frequency may have originated from the improved efficiency of the modified crRNA. It is important to mention that a more careful identification of potential off-target sites is necessary, and accordingly, targets with off-target sites showing high sequence similarities should be avoided through *in silico* analysis. Then, highly specific and efficient genome engineering can be achieved via 3′-U-rich crRNA with ribosyl-2′-O-methylation in the CRISPR-Cas12a system.

## Discussion

It has been more than four years since Zhang and colleagues first introduced the CRISPR-Cas12a system^5^; since then Cas12a has been proposed to provide multiple advantages over Cas9 for genome editing purposes. First, Cas12a has a smaller gene size than most Cas9 analogs, which is a critical element for viral transduction of genes. Moreover, the guide RNA for Cas12a (crRNA) is less than half the size of the sgRNA for Cas9, which makes it much easier to synthesize the crRNA with high purity. Additionally, credible research articles suggest that Cpf1 has higher specificity than Cas9^36^. These traits are critical for clinical applications of programmable nucleases. Nonetheless, the adoption of Cas12a has been less enthusiastic than expected. The paucity of research articles searchable in PubMed reflects this notion; indeed, almost 12,000 CRISPR-Cas9-related research articles were deposited in PubMed in the 4 years after the first publication in Science in January 2013. In contrast, only 400 or so papers concerning Cas12a were published in the same period of time. Moreover, many more studies on Cas9 than on Cas12a have been published during the same period (October 2015–present). We propose that this startlingly low adoption of Cas12a stems mainly, if not exclusively, from its low efficiency compared with that of Cas9. Thus, improving the efficiency of Cas12a is a critical factor for making the best use of the inherent advantages of Cas12a over Cas9. Unlike the guide RNA for Cas9, the crRNA for Cpf1 has a 5′-oriented direct repeat sequence and a target-complementary sequence located in the 3’ region. These features suggest that there is room for engineering of the crRNA at the 3′-end. Previously, we observed that 3′-terminal addition of multiple uridine bases to the crRNA significantly improved Cas12a indel efficiency. In this study, we further engineered Cas12a crRNA by modifying the U-rich 3′-overhang with 2′-O-methylation. This modification improved the safety and efficiency of genome engineering with the CRISPR-Cas12a system and is expected to facilitate the generation of various animal models. Moreover, the engineered crRNA was evaluated to add a powerful tool to the genome editing toolbox.

Ribonucleoprotein (RNP) complexes have been suggested to increase the specificity of gene editing^26^, and guide RNA modifications can have synergistic effects on specificity. 3′-Phosphorothioate linkage, either alone or together with 2′-O-ribose modifications, confers Cas9 with significantly improved target specificity^21^. It is interesting to note that an RNA-DNA hybrid guide exhibits enhanced specificity^37^. In particular, highly specific editing has been achieved by bridged nucleic acids (BNAs) in which the 2′-O and 4′-C atoms of the ribose are joined through a methylene bridge. These BNAs include N-methyl-substituted 2′,4′-bridged ribose (BNA^NC^[N-Me]) and S-constrained ethyl ribose^22^. OM U-rich crRNA was responsible for further improvement of the genome editing efficiency of Cas12a, but the improved efficiency led to undesirable off-target effects at targets with high sequence similarity that were tolerant of single mismatches (Table 2). If this drawback is addressed, the engineered Cas12a system will yield unsurpassed high genome editing performance, as is required in clinical settings. Further applications of chemical modifications that are validated to improve specificity, including BNAs, will help achieve more advanced genome editing in terms of specificity and safety.

## Supplementary information

Supplementary material
