# Supplementary material for: Highly efficient and safe genome editing by CRISPR-Cas12a using CRISPR RNA with a ribosyl-2′-O-methylated uridinylate-rich 3′-overhang in mouse zygotes
Source: Exp Mol Med. 2020 Nov 9;52(11):1823–30. doi: 10.1038/s12276-020-00521-7 (PMC8080787; doi:10.1038/s12276-020-00521-7)
Supplement: Supplementary file 1 — Supplementary material [file 12276_2020_521_MOESM1_ESM.pptx]

## Slide 1
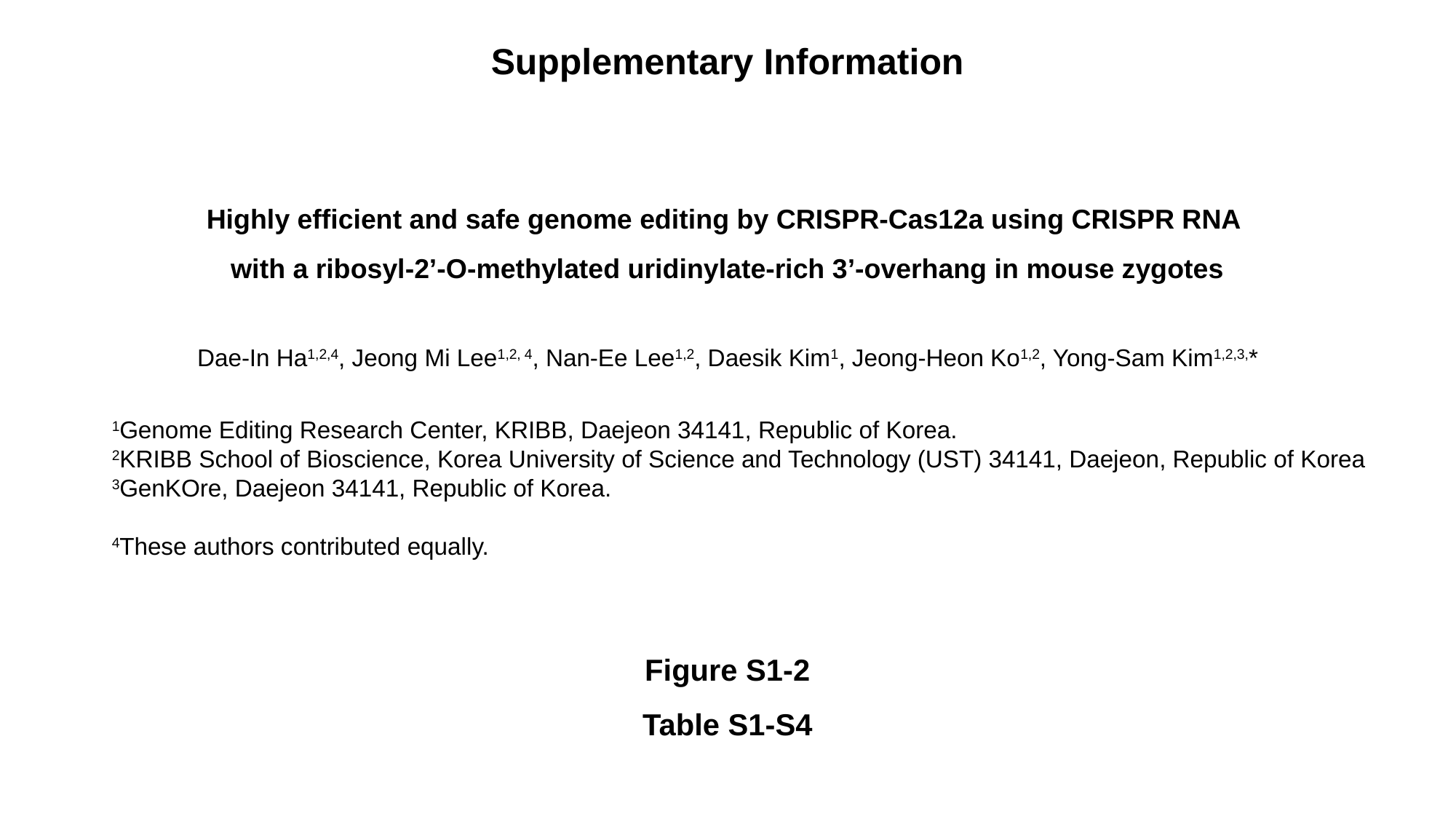

Supplementary Information
Highly efficient and safe genome editing by CRISPR-Cas12a using CRISPR RNA
with a ribosyl-2’-O-methylated uridinylate-rich 3’-overhang in mouse zygotes
Dae-In Ha1,2,4, Jeong Mi Lee1,2, 4, Nan-Ee Lee1,2, Daesik Kim1, Jeong-Heon Ko1,2, Yong-Sam Kim1,2,3,*
1Genome Editing Research Center, KRIBB, Daejeon 34141, Republic of Korea.
2KRIBB School of Bioscience, Korea University of Science and Technology (UST) 34141, Daejeon, Republic of Korea
3GenKOre, Daejeon 34141, Republic of Korea.
4These authors contributed equally.
Figure S1-2
Table S1-S4

## Slide 2
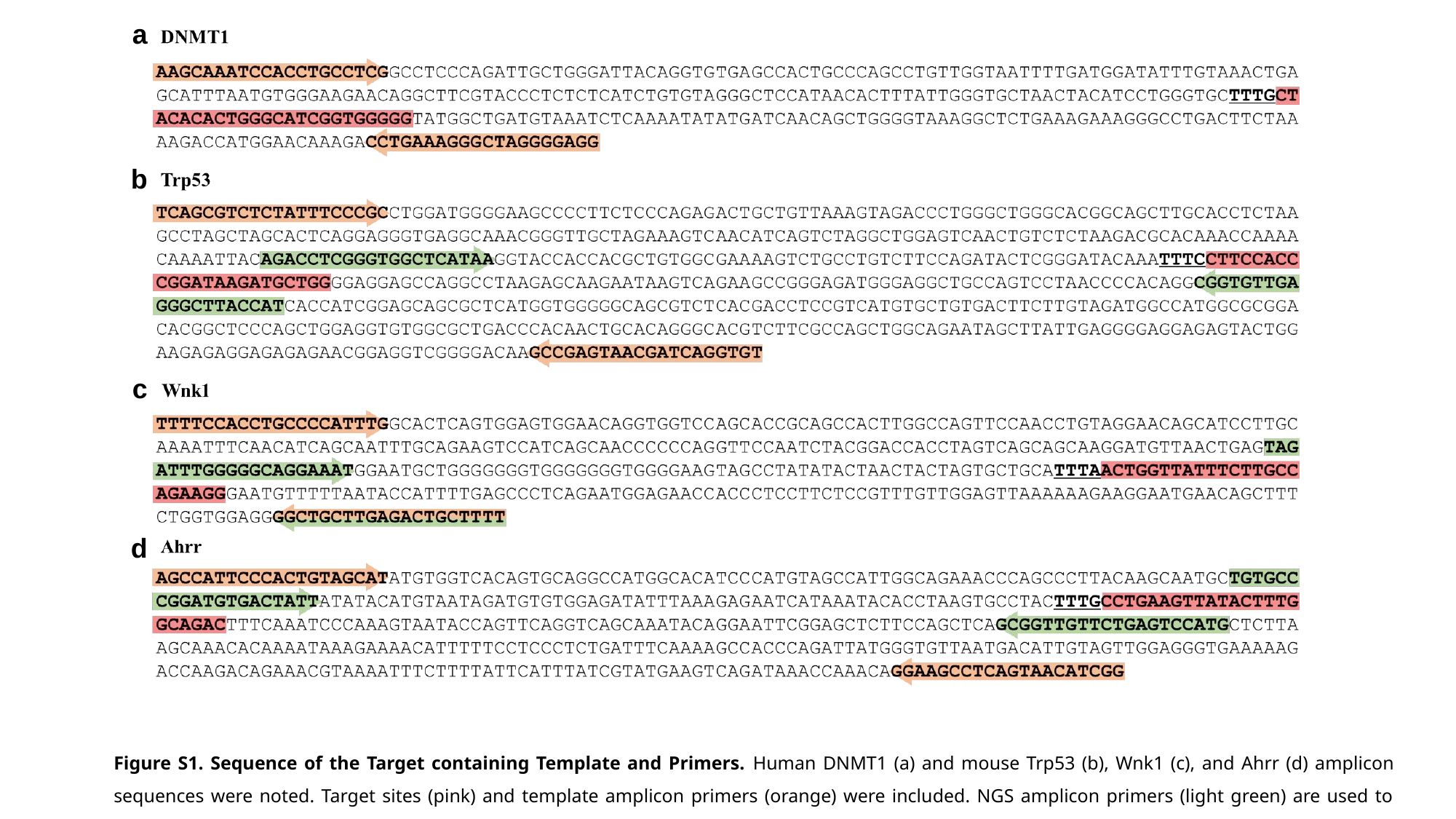

a
b
c
d
Figure S1. Sequence of the Target containing Template and Primers. Human DNMT1 (a) and mouse Trp53 (b), Wnk1 (c), and Ahrr (d) amplicon sequences were noted. Target sites (pink) and template amplicon primers (orange) were included. NGS amplicon primers (light green) are used to amplify templates within an analytical size range.

## Slide 3
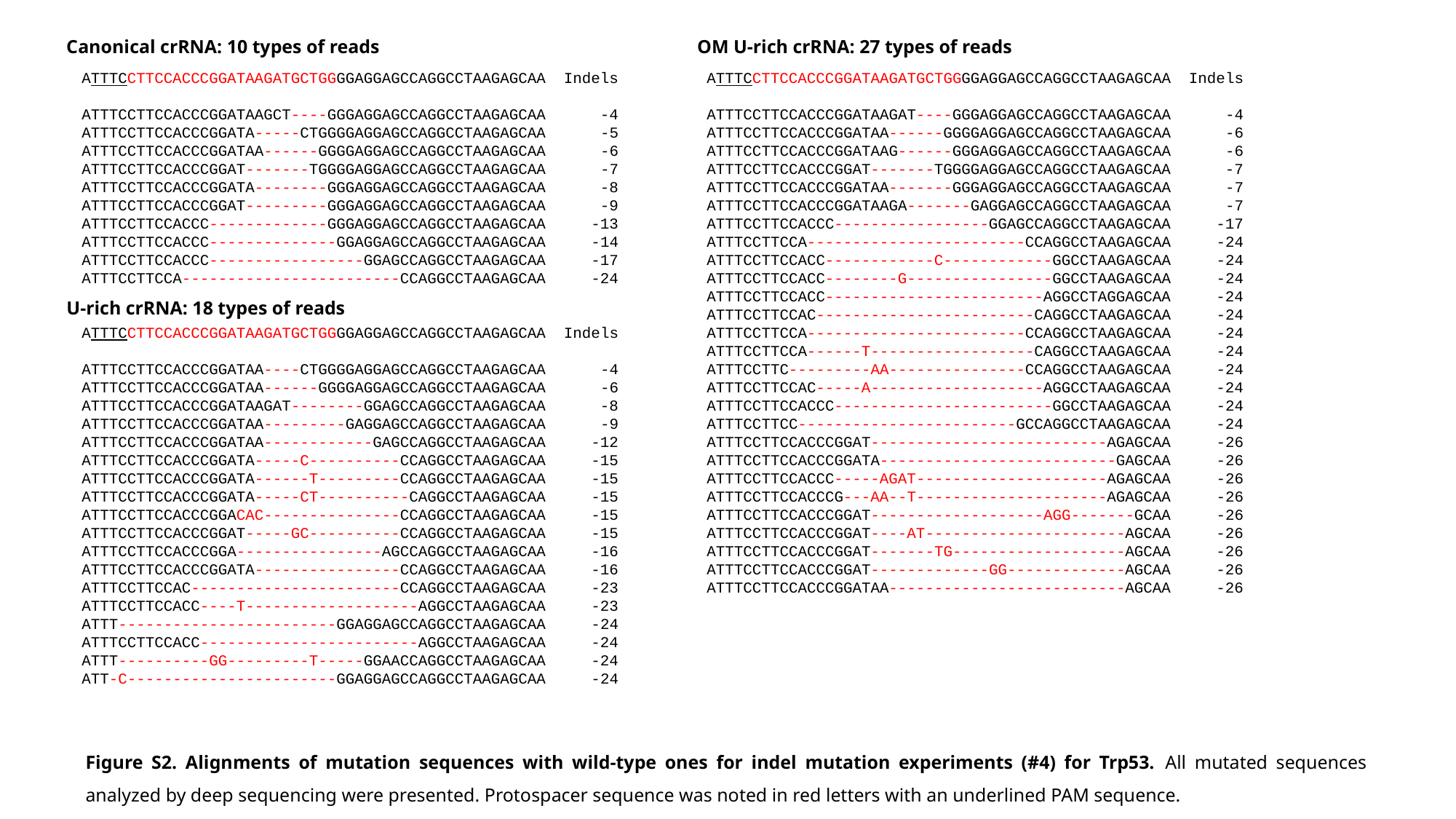

Canonical crRNA: 10 types of reads
OM U-rich crRNA: 27 types of reads
ATTTCCTTCCACCCGGATAAGATGCTGGGGAGGAGCCAGGCCTAAGAGCAA Indels
ATTTCCTTCCACCCGGATAAGCT----GGGAGGAGCCAGGCCTAAGAGCAA -4
ATTTCCTTCCACCCGGATA-----CTGGGGAGGAGCCAGGCCTAAGAGCAA -5
ATTTCCTTCCACCCGGATAA------GGGGAGGAGCCAGGCCTAAGAGCAA -6
ATTTCCTTCCACCCGGAT-------TGGGGAGGAGCCAGGCCTAAGAGCAA -7
ATTTCCTTCCACCCGGATA--------GGGAGGAGCCAGGCCTAAGAGCAA -8
ATTTCCTTCCACCCGGAT---------GGGAGGAGCCAGGCCTAAGAGCAA -9
ATTTCCTTCCACCC-------------GGGAGGAGCCAGGCCTAAGAGCAA -13
ATTTCCTTCCACCC--------------GGAGGAGCCAGGCCTAAGAGCAA -14
ATTTCCTTCCACCC-----------------GGAGCCAGGCCTAAGAGCAA -17
ATTTCCTTCCA------------------------CCAGGCCTAAGAGCAA -24
ATTTCCTTCCACCCGGATAAGATGCTGGGGAGGAGCCAGGCCTAAGAGCAA Indels
ATTTCCTTCCACCCGGATAA----CTGGGGAGGAGCCAGGCCTAAGAGCAA -4
ATTTCCTTCCACCCGGATAA------GGGGAGGAGCCAGGCCTAAGAGCAA -6
ATTTCCTTCCACCCGGATAAGAT--------GGAGCCAGGCCTAAGAGCAA -8
ATTTCCTTCCACCCGGATAA---------GAGGAGCCAGGCCTAAGAGCAA -9
ATTTCCTTCCACCCGGATAA------------GAGCCAGGCCTAAGAGCAA -12
ATTTCCTTCCACCCGGATA-----C----------CCAGGCCTAAGAGCAA -15
ATTTCCTTCCACCCGGATA------T---------CCAGGCCTAAGAGCAA -15
ATTTCCTTCCACCCGGATA-----CT----------CAGGCCTAAGAGCAA -15
ATTTCCTTCCACCCGGACAC---------------CCAGGCCTAAGAGCAA -15
ATTTCCTTCCACCCGGAT-----GC----------CCAGGCCTAAGAGCAA -15
ATTTCCTTCCACCCGGA----------------AGCCAGGCCTAAGAGCAA -16
ATTTCCTTCCACCCGGATA----------------CCAGGCCTAAGAGCAA -16
ATTTCCTTCCAC-----------------------CCAGGCCTAAGAGCAA -23
ATTTCCTTCCACC----T-------------------AGGCCTAAGAGCAA -23
ATTT------------------------GGAGGAGCCAGGCCTAAGAGCAA -24
ATTTCCTTCCACC------------------------AGGCCTAAGAGCAA -24
ATTT----------GG---------T-----GGAACCAGGCCTAAGAGCAA -24
ATT-C-----------------------GGAGGAGCCAGGCCTAAGAGCAA -24
ATTTCCTTCCACCCGGATAAGATGCTGGGGAGGAGCCAGGCCTAAGAGCAA Indels
ATTTCCTTCCACCCGGATAAGAT----GGGAGGAGCCAGGCCTAAGAGCAA -4
ATTTCCTTCCACCCGGATAA------GGGGAGGAGCCAGGCCTAAGAGCAA -6
ATTTCCTTCCACCCGGATAAG------GGGAGGAGCCAGGCCTAAGAGCAA -6
ATTTCCTTCCACCCGGAT-------TGGGGAGGAGCCAGGCCTAAGAGCAA -7
ATTTCCTTCCACCCGGATAA-------GGGAGGAGCCAGGCCTAAGAGCAA -7
ATTTCCTTCCACCCGGATAAGA-------GAGGAGCCAGGCCTAAGAGCAA -7
ATTTCCTTCCACCC-----------------GGAGCCAGGCCTAAGAGCAA -17
ATTTCCTTCCA------------------------CCAGGCCTAAGAGCAA -24
ATTTCCTTCCACC------------C------------GGCCTAAGAGCAA -24
ATTTCCTTCCACC--------G----------------GGCCTAAGAGCAA -24
ATTTCCTTCCACC------------------------AGGCCTAGGAGCAA -24
ATTTCCTTCCAC------------------------CAGGCCTAAGAGCAA -24
ATTTCCTTCCA------------------------CCAGGCCTAAGAGCAA -24
ATTTCCTTCCA------T------------------CAGGCCTAAGAGCAA -24
ATTTCCTTC---------AA---------------CCAGGCCTAAGAGCAA -24
ATTTCCTTCCAC-----A-------------------AGGCCTAAGAGCAA -24
ATTTCCTTCCACCC------------------------GGCCTAAGAGCAA -24
ATTTCCTTCC------------------------GCCAGGCCTAAGAGCAA -24
ATTTCCTTCCACCCGGAT--------------------------AGAGCAA -26
ATTTCCTTCCACCCGGATA--------------------------GAGCAA -26
ATTTCCTTCCACCC-----AGAT---------------------AGAGCAA -26
ATTTCCTTCCACCCG---AA--T---------------------AGAGCAA -26
ATTTCCTTCCACCCGGAT-------------------AGG-------GCAA -26
ATTTCCTTCCACCCGGAT----AT----------------------AGCAA -26
ATTTCCTTCCACCCGGAT-------TG-------------------AGCAA -26
ATTTCCTTCCACCCGGAT-------------GG-------------AGCAA -26
ATTTCCTTCCACCCGGATAA--------------------------AGCAA -26
U-rich crRNA: 18 types of reads
Figure S2. Alignments of mutation sequences with wild-type ones for indel mutation experiments (#4) for Trp53. All mutated sequences analyzed by deep sequencing were presented. Protospacer sequence was noted in red letters with an underlined PAM sequence.

## Slide 4
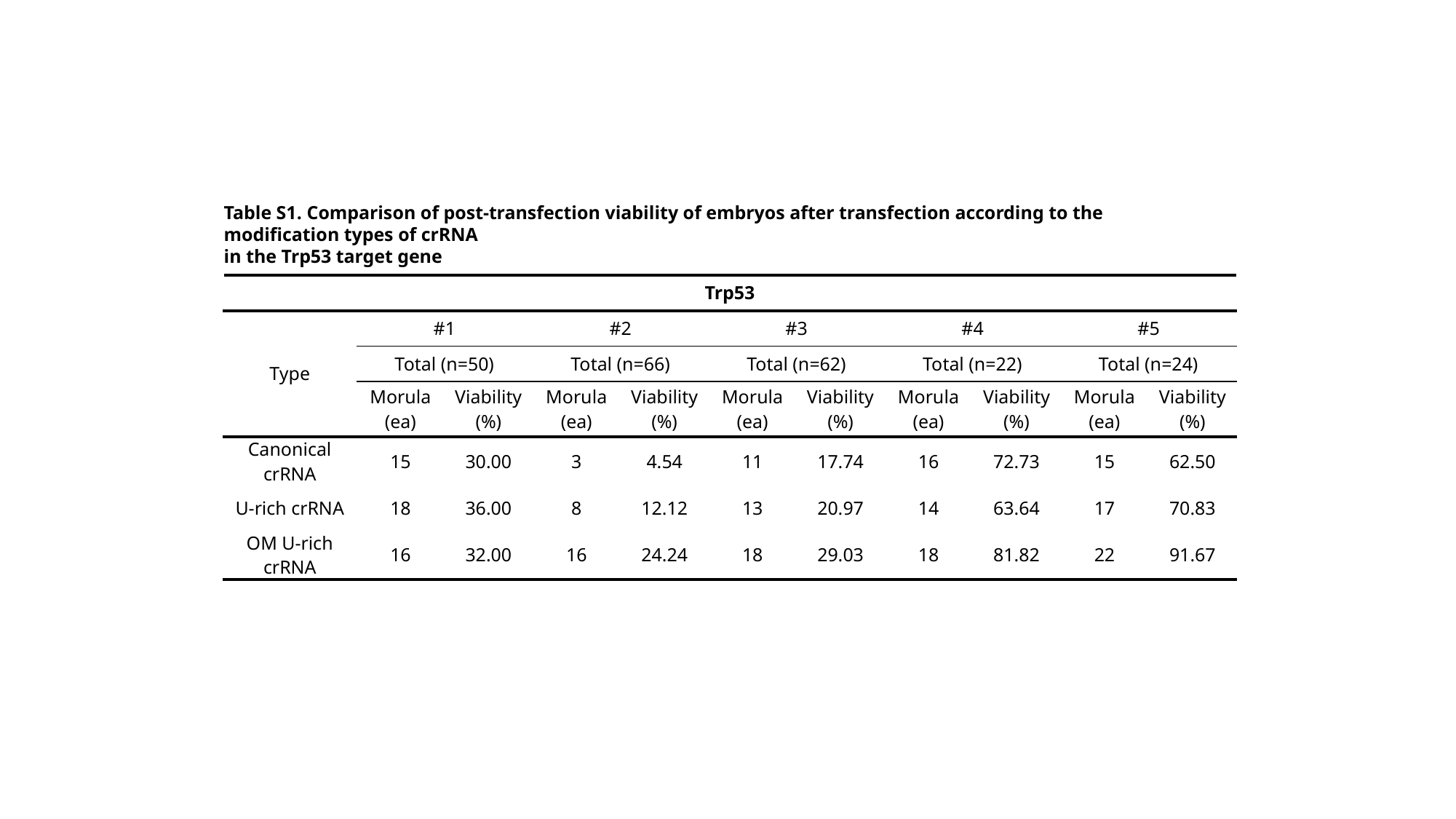

Table S1. Comparison of post-transfection viability of embryos after transfection according to the modification types of crRNA
in the Trp53 target gene
| Trp53 | | | | | | | | | | |
| --- | --- | --- | --- | --- | --- | --- | --- | --- | --- | --- |
| Type | #1 | | #2 | | #3 | | #4 | | #5 | |
| | Total (n=50) | | Total (n=66) | | Total (n=62) | | Total (n=22) | | Total (n=24) | |
| | Morula (ea) | Viability (%) | Morula (ea) | Viability (%) | Morula (ea) | Viability (%) | Morula (ea) | Viability (%) | Morula (ea) | Viability (%) |
| Canonical crRNA | 15 | 30.00 | 3 | 4.54 | 11 | 17.74 | 16 | 72.73 | 15 | 62.50 |
| U-rich crRNA | 18 | 36.00 | 8 | 12.12 | 13 | 20.97 | 14 | 63.64 | 17 | 70.83 |
| OM U-rich crRNA | 16 | 32.00 | 16 | 24.24 | 18 | 29.03 | 18 | 81.82 | 22 | 91.67 |

## Slide 5
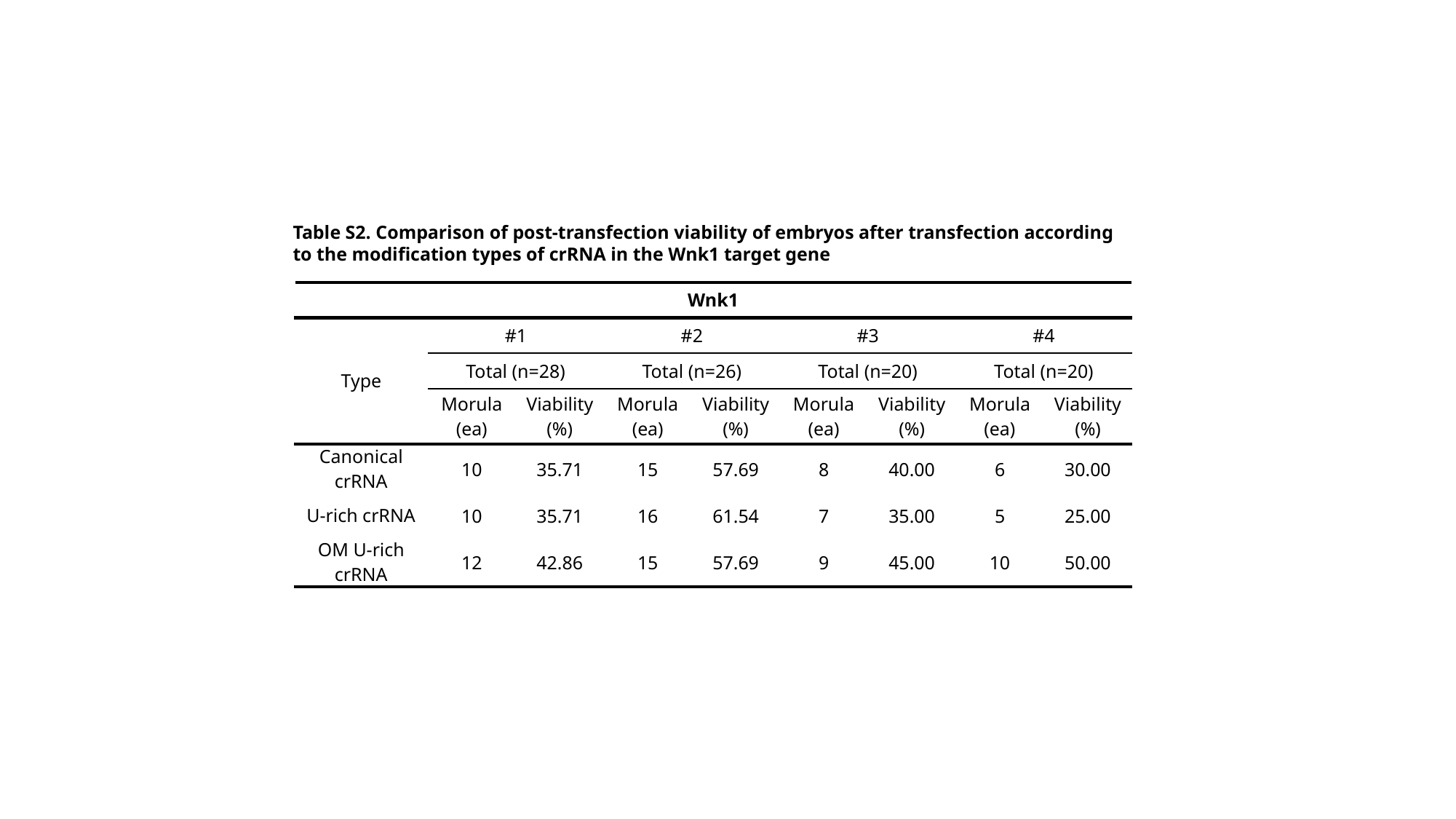

Table S2. Comparison of post-transfection viability of embryos after transfection according to the modification types of crRNA in the Wnk1 target gene
| Wnk1 | | | | | | | | |
| --- | --- | --- | --- | --- | --- | --- | --- | --- |
| Type | #1 | | #2 | | #3 | | #4 | |
| | Total (n=28) | | Total (n=26) | | Total (n=20) | | Total (n=20) | |
| | Morula (ea) | Viability (%) | Morula (ea) | Viability (%) | Morula (ea) | Viability (%) | Morula (ea) | Viability (%) |
| Canonical crRNA | 10 | 35.71 | 15 | 57.69 | 8 | 40.00 | 6 | 30.00 |
| U-rich crRNA | 10 | 35.71 | 16 | 61.54 | 7 | 35.00 | 5 | 25.00 |
| OM U-rich crRNA | 12 | 42.86 | 15 | 57.69 | 9 | 45.00 | 10 | 50.00 |

## Slide 6
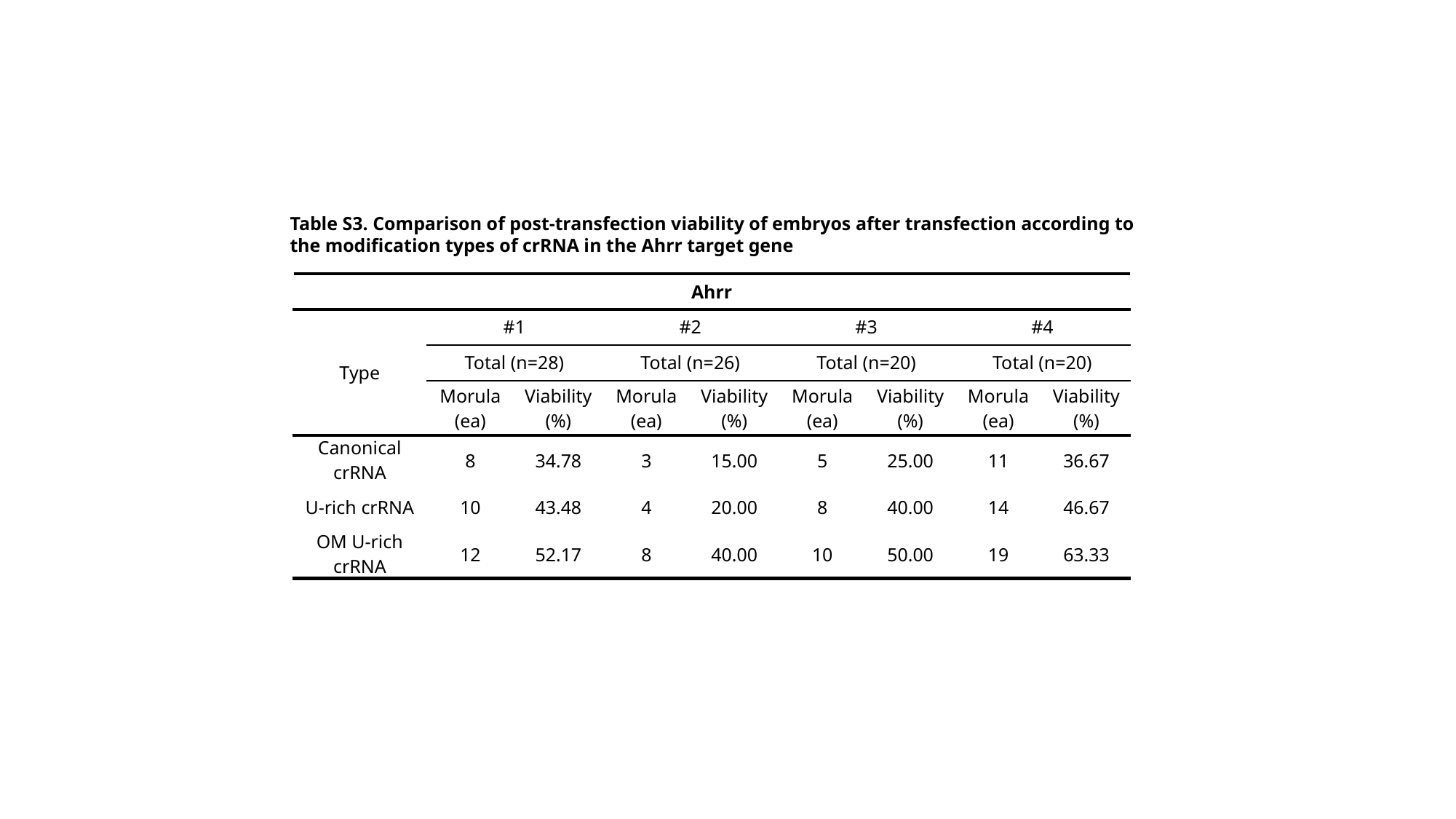

Table S3. Comparison of post-transfection viability of embryos after transfection according to the modification types of crRNA in the Ahrr target gene
| Ahrr | | | | | | | | |
| --- | --- | --- | --- | --- | --- | --- | --- | --- |
| Type | #1 | | #2 | | #3 | | #4 | |
| | Total (n=28) | | Total (n=26) | | Total (n=20) | | Total (n=20) | |
| | Morula (ea) | Viability (%) | Morula (ea) | Viability (%) | Morula (ea) | Viability (%) | Morula (ea) | Viability (%) |
| Canonical crRNA | 8 | 34.78 | 3 | 15.00 | 5 | 25.00 | 11 | 36.67 |
| U-rich crRNA | 10 | 43.48 | 4 | 20.00 | 8 | 40.00 | 14 | 46.67 |
| OM U-rich crRNA | 12 | 52.17 | 8 | 40.00 | 10 | 50.00 | 19 | 63.33 |

## Slide 7
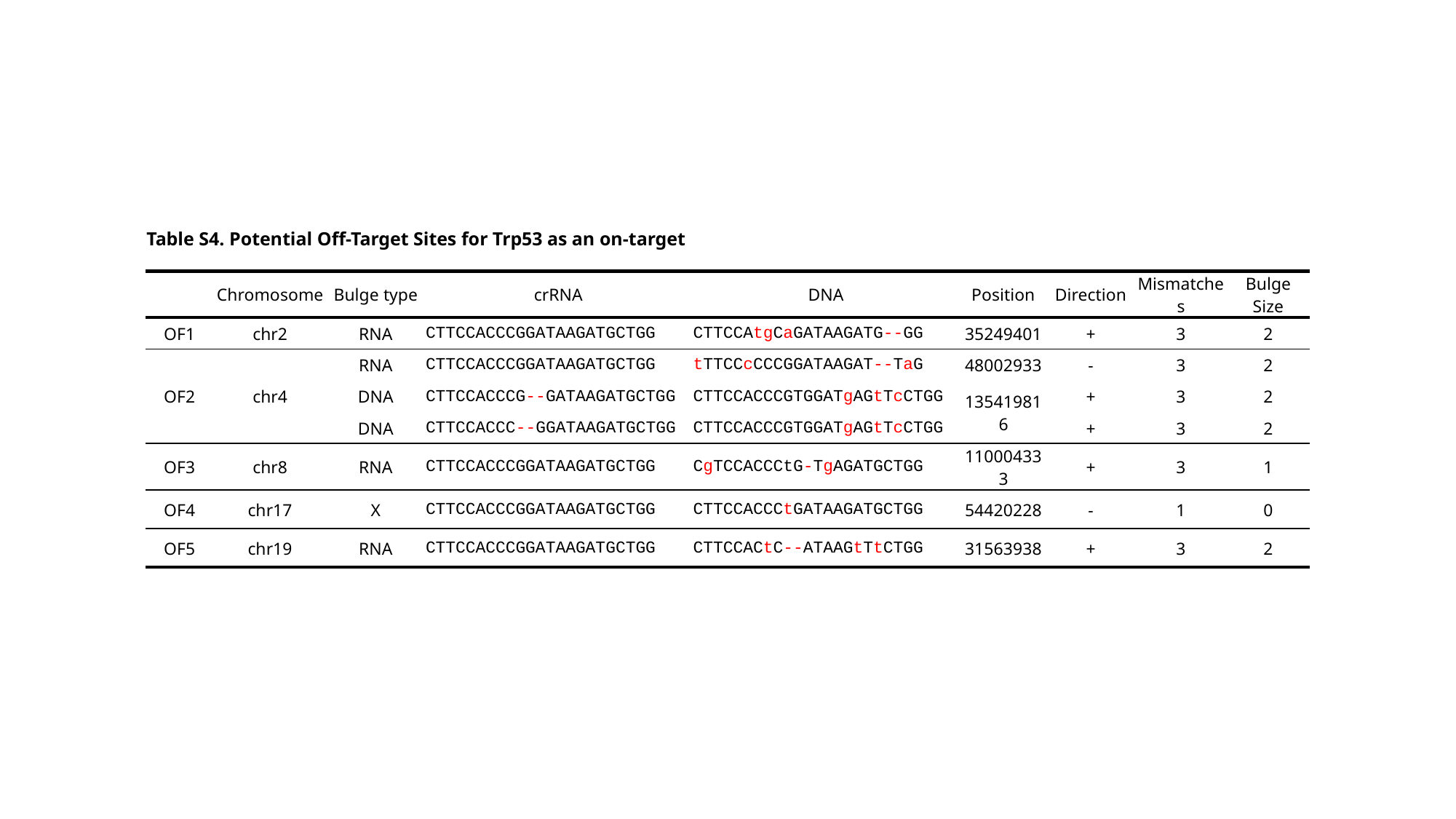

Table S4. Potential Off-Target Sites for Trp53 as an on-target
| | Chromosome | Bulge type | crRNA | DNA | Position | Direction | Mismatches | Bulge Size |
| --- | --- | --- | --- | --- | --- | --- | --- | --- |
| OF1 | chr2 | RNA | CTTCCACCCGGATAAGATGCTGG | CTTCCAtgCaGATAAGATG--GG | 35249401 | + | 3 | 2 |
| OF2 | chr4 | RNA | CTTCCACCCGGATAAGATGCTGG | tTTCCcCCCGGATAAGAT--TaG | 48002933 | - | 3 | 2 |
| | | DNA | CTTCCACCCG--GATAAGATGCTGG | CTTCCACCCGTGGATgAGtTcCTGG | 135419816 | + | 3 | 2 |
| | | DNA | CTTCCACCC--GGATAAGATGCTGG | CTTCCACCCGTGGATgAGtTcCTGG | | + | 3 | 2 |
| OF3 | chr8 | RNA | CTTCCACCCGGATAAGATGCTGG | CgTCCACCCtG-TgAGATGCTGG | 110004333 | + | 3 | 1 |
| OF4 | chr17 | X | CTTCCACCCGGATAAGATGCTGG | CTTCCACCCtGATAAGATGCTGG | 54420228 | - | 1 | 0 |
| OF5 | chr19 | RNA | CTTCCACCCGGATAAGATGCTGG | CTTCCACtC--ATAAGtTtCTGG | 31563938 | + | 3 | 2 |
